# Supplementary material for: Absence of internal multidecadal and interdecadal oscillations in climate model simulations
Source: Nat Commun. 2020 Jan 3;11:49. doi: 10.1038/s41467-019-13823-w (PMC6941994; doi:10.1038/s41467-019-13823-w)
Supplement: Supplementary file 1 — Supplementary Information [file 41467_2019_13823_MOESM1_ESM.pdf]

Supplementary Information

Absence of Internal Multidecadal and Interdecadal Oscillations in Climate Model Simulations

Mann et al.

Supplementary Table 1. CMIP5 Climate Model Simulations

Model	Number of Realizations	1st *and* 2nd aerosol indirect effects	Length of Simulations (years)
a. Historical Simulations			
ACCESS1-0	1	1	156
ACCESS1-3	3	1	156
bcc-csm1-1	3	0	162
bcc-csm1-1-m	3	0	162
BNU-ESM	1	0	155
CanESM2	5	0	155
CCSM4	6	0	155
CESM1-BGC	1	0	155
CESM1-CAM5	3	1	155
CESM1-FASTCHEM	3	0	155
CESM1-WACCM	1	0	155
CMCC-CESM	1	0	156
CMCC-CM	1	0	156
CMCC-CMS	1	0	156
CNRM-CM5	10	0	156
CNRM-CM5-2	1	0	156
CSIRO-Mk3-6-0	10	1	155
FGOALS-g2	4	1	156
FIO-ESM	3	0	155
GFDL-CM2.1	10	0	145
GFDL-CM3	5	1	145
GFDL-ESM2G	1	0	144
GFDL-ESM2M	1	0	144
GISS-E2-H p1	6	0	156
GISS-E2-H p2	5	0	156
GISS-E2-H p3	6	0	156
GISS-E2-H-CC	1	0	160
GISS-E2-R p1	6	0	155
GISS-E2-R p2	6	0	156
GISS-E2-R p3	6	0	156
GISS-E2-R p100	6	0	156
GISS-E2-R-CC	1	0	160
HadCM3	10	0	143
HadGEM2-AO	1	1	143
HadGEM2-CC	1	1	143
HadGEM2-ES	5	1	143
INMCM4	1	0	155
IPSL-CM5A-LR	6	0	155
IPSL-CM5A-MR	3	0	155
IPSL-CM5B-LR	1	0	155
MIROC-ESM	3	1	156
MIROC-ESM-CHEM	1	1	156
MPI-ESM-LR	2	0	156
MRI-CGCM3 p1	3	1	156
MRI-CGCM3 p2	1	1	156
MRI-ESM1	1	1	155
NorESM1-M	3	1	155
NorESM1-ME	1	1	155
b. Anthropogenic Only Simulation			
CCSM4 p11	4	–	155
CESM1-CAM5 p11	3	–	155
CNRM-CM5	10	–	163
GFDL-CM3 p2	3	–	145
GFDL-ESM2M p2	1	–	144
GISS-E2-H p109	3	–	163
GISS-E2-H p309	3	–	163
GISS-E2-R p109	5	–	163
GISS-E2-R p309	5	–	163
IPSL-CM5A-LR p2	3	–	155

Supplementary Table 1. (continued). CMIP5 Climate Model Simulations

Model	Number of Realizations	1st *and* 2nd aerosol indirect effects	Length of Simulations (years)
c.	Pre-Industrial Control Simulations		
ACCESS 1-0	1	–	500
ACCESS 1-3	1	–	500
BNU-ESM	1	–	558
CCSM4	3	–	119-1051
CESM1-BGC	1	–	500
CESM1-CAM5	1	–	318
CESM1-FASTCHEM	1	–	221
CESM1-WACCM	1	–	199
CMCC-CESM	1	–	277
CMCC-CM	1	–	500
CMCC-CMS	1	–	850
CNRM-CM5	1	–	359
CNRM-CM5-2 p1	1	–	140
CNRM-CM5-2 p2	1	–	140
CSIRO-Mk3-6-0	1	–	499
CSIRO-Mk3L-1-2	1	–	999
FGOALS-g2	1	–	700
FGOALS-s2	1	–	500
FIO-ESM	1	–	799
GFDL-CM3	1	–	499
GFDL-ESM2G	1	–	499
GFDL-ESM2M	1	–	499
GISS-E2-H p1	1	–	1770
GISS-E2-H p2	1	–	531
GISS-E2-H p3	1	–	531
GISS-E2-H-CC	1	–	251
GISS-E2-R p1	1	–	1163
GISS-E2-R p2	1	–	100
GISS-E2-R p3	1	–	5875
GISS-E2-R p141	1	–	531
GISS-E2-R p142	1	–	531
GISS-E2-R-CC	1	–	251
HadCM3	1	–	1183
HadGEM2-AO	1	–	698
HadGEM2-CC	1	–	236
HadGEM2-ES	1	–	566
INMCM4	1	–	499
MIROC-ESM	1	–	630
MIROC-ESM-CHEM	1	–	255
MIROC4h	1	–	99
MIROC5	1	–	869
MPI-ESM-LR	1	–	1000
MPI-ESM-MR	1	–	1000
MPI-ESM-p	1	–	1156
MRI-CGCM3 p1	1	–	500

Supplementary Table 1. Model simulations used in this study: (a) CMIP5 All-Forcing Historical Simulations, (b) CMIP5 Historical Simulations only using Anthropogenic Forcing and (c) CMIP5 Pre-Industrial Control Simulations, specifying the number of simulations as well as whether the model contains aerosol indirect effects (for all-forcing simulations: 1 – yes, 0 – no).

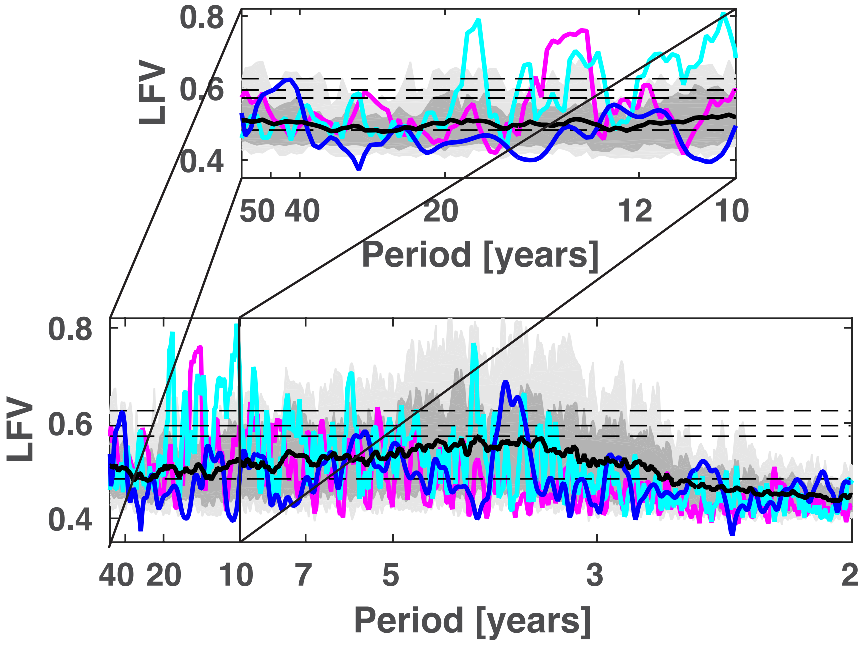

Supplementary Figure 1. MTM-SVD Spectra of Historical CMIP5 control simulations (shading with mean over all simulations shown by black curve) and historical temperature observations (blue), along with the spectra for the HadGEM2-ES (purple) and MPI-ESM-LR (cyan) simulations. Conventions as in Figure 1.

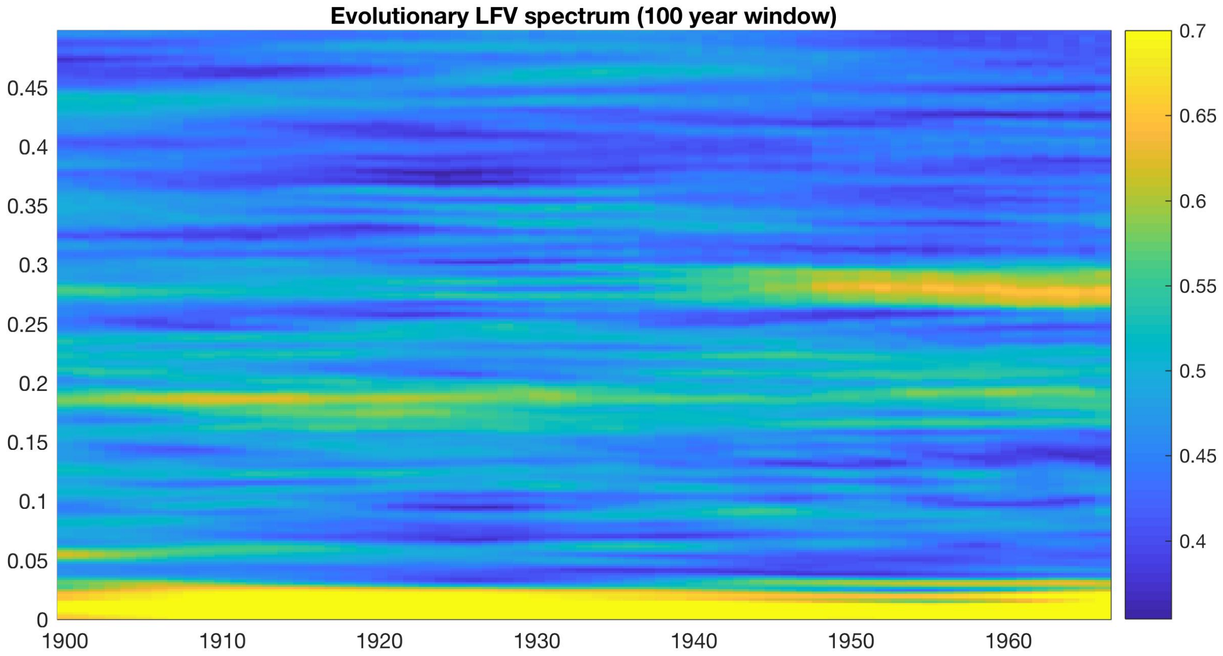

Supplementary Figure 2. Evolutionary MTM-SVD Spectra of Historical surface temperature observations using a 100 year moving window from 1850 to 2018 (year indicated represents center of the 100 year moving window)

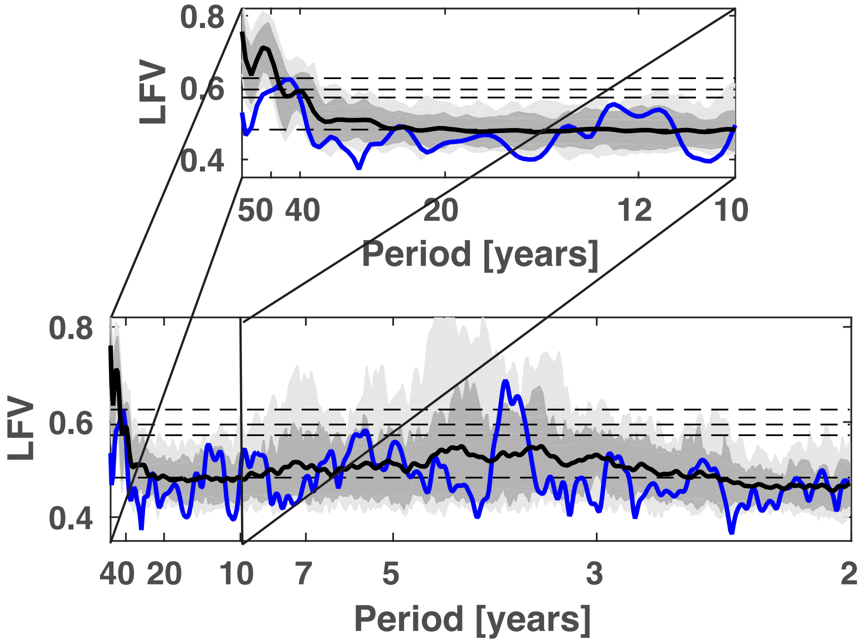

Supplementary Figure 3. Spectra for the global surface temperature fields from historical CMIP5 simulations using anthropogenic forcing only and historical observations. Shading with mean over all model simulations is shown by black curve and historical result is shown by blue curve. Lower (f=0.015 cycle/year) and upper (f=0.5 cycle/year) bound on frequencies shown correspond to edge of secular band and Nyquist sampling frequency. Inset zooms in on the decadal (f=0.1 cycle/year) and longer periodicities. Horizontal dashed lines correspond to median (p=0.5) and p=0.1, 0.05 and 0.01 significance levels relative to coloured noise null hypothesis. Local Fractional Variance (LFV).
